# Mitochondrial Dysfunction in Alzheimer’s Disease and the Rationale for Bioenergetics Based Therapies

**DOI:** 10.14336/AD.2015.1007

**Published:** 2016-03-15

**Authors:** Isaac G. Onyango, Jameel Dennis, Shaharyah M. Khan

**Affiliations:** Gencia Biotechnology, 706 B Forest St, Charlottesville, VA 22903, USA; Gencia Biotechnology, 706 B Forest St, Charlottesville, VA 22903, USA; Gencia Biotechnology, 706 B Forest St, Charlottesville, VA 22903, USA

**Keywords:** Alzheimer’s disease, mitochondria, mitophagy, oxidative stress, neuroinflammation, mitochondrial biogenesis, neuroinflammation

## Abstract

Alzheimer’s disease (AD) is a debilitating neurodegenerative disorder characterized by the progressive loss of cholinergic neurons, leading to the onset of severe behavioral, motor and cognitive impairments. It is a pressing public health problem with no effective treatment. Existing therapies only provide symptomatic relief without being able to prevent, stop or reverse the pathologic process. While the molecular basis underlying this multifactorial neurodegenerative disorder remains a significant challenge, mitochondrial dysfunction appears to be a critical factor in the pathogenesis of this disease. It is therefore important to target mitochondrial dysfunction in the prodromal phase of AD to slow or prevent the neurodegenerative process and restore neuronal function. In this review, we discuss mechanisms of action and translational potential of current mitochondrial and bioenergetic therapeutics for AD including: mitochondrial enhancers to potentiate energy production; antioxidants to scavenge reactive oxygen species and reduce oxidative damage; glucose metabolism and substrate supply; and candidates that target apoptotic and mitophagy pathways to remove damaged mitochondria. While mitochondrial therapeutic strategies have shown promise at the preclinical stage, there has been little progress in clinical trials thus far.

Alzheimer's disease (AD) is the most common form of dementia and affects millions worldwide. It is characterized by severe memory loss, with episodic memory being particularly impaired during the initial phases. Most AD cases occur sporadically, although inheritance of certain susceptibility genes enhances risk. A role for dysfunctional mitochondria in AD pathogenesis has been postulated [1-3].

Cumulative evidence reveal that the regulation of mitochondrial turnover and function becomes impaired as a function of age in the brain and may contribute to neurodegeneration in AD [4]. Cerebral hypometabolism is evident in affected brain regions [5, 6] where mitochondrial structure is altered [7, 8]. The expression and activity of mitochondrial enzymes important for metabolism, including cytochrome *c* oxidase (COX), α-ketoglutarate dehydrogenase complex, and pyruvate dehydrogenase complex is reduced [9-11]. AD brain mitochondria have reduced membrane potential, increased permeability, and produce excess reactive oxygen species (ROS) which damages proteins, lipids, and nucleic acids, and are believed to contribute to the pathogenesis of neurodegeneration. Growing evidence suggest that elevated amyloid-β (Aβ) levels contribute to the mitochondrial abnormalities and although the mechanism is not clearly established, both amyloid precursor protein (APP) and Aβ are found in mitochondrial membranes and interact with mitochondrial proteins. Overproduction of the APP and Aβ may affect dynamics of mitochondrial fusion/fission [12-14], impair mitochondrial transport, disrupt the electron transfer chain, increase ROS production [15-17], and impair mitochondrial function [18-20]. These findings build a strong case for mitochondrial dysfunction in AD and effective treatment will likely include targets that address mitochondrial function [21-23].

## Mitochondrial Biogenesis

Mitochondrial biogenesis plays an essential role in maintaining an adequate functional neuronal mitochondrial mass by compensating for damaged mitochondria that have been eliminated. It is highly regulated and requires coordination and crosstalk between the nuclear and mitochondrial genomes [24]. While mitochondrial biogenesis occurs on a regular basis in healthy cells where mitochondria constantly divide and fuse with each other [25-27]; it also occurs in response to oxidative stress, increased energy demand, exercise training and certain diseases. The status of mitochondrial biogenesis in AD neurons is unclear [28]. Biogenesis is induced by the peroxisome proliferator-activated receptor γ coactivator-1α (PGC-1α) which activates different transcription factors, including nuclear respiratory factors 1 and 2 proteins (NRF-1 and NRF-2) and the mitochondrial transcription factor A (TFAM) [4, 29, 30];[28]. NRF-1 and NRF-2 regulate transcription of nuclear and mitochondrial genes involved in OXPHOS, electron transport (complex I-V), mtDNA transcription/replication, heme biosynthesis, protein import/assembly, ion channels, shuttles, and translation [31].

NRF-1 or NRF-2 also contribute to expression of nuclear encoded genes involved in biogenesis including [32]factor A (TFAM), mitochondrial transcription factor B1 or B2 (TFB1M or TFB2M), and mitochondrial RNA polymerase (POLRMT), and mitochondrial transcription termination factor (MTERF), mitochondrial DNA helicase (TWINKLE), single-stranded DNA-binding protein (mtSSB), and POLγB [33] [34] but not POLγA and MTERF3 [34]. When newly formed daughter mitochondria have been incorporated into the mitochondrial network, mitochondria that have been damaged or that have lost membrane potential are specifically targeted for degradation via an autophagy-like process termed as mitophagy [35, 36]. Mitochondrial biogenesis is thought to be impaired in AD where the quantity of mitochondria as well as levels of NRF 1, NRF 2, and TFAM along with nuclear levels of PGC-1α are reduced in hippocampal tissues from AD brain compared to age matched control brain [37] [38] [39].

PGC-1α activity at both the transcriptional and post-translational level is modulated by the nutrient supply and energy balance within the cell [40] and mitochondrial damage [41] [42]. Furthermore, PPARs, mTOR (acting on YY1), and CREB (downstream of PKA signaling) transcriptionally activate PGC-1α to initiate mitochondrial biogenesis [40]. At the post-translational level, PGC-1α is regulated by both phosphorylation and acetylation events. AMP-dependent kinase (AMPK) can phosphorylate and activate PGC-1α while GCN5-mediated acetylation inhibits PGC-1α activity [40]. Deacetylation of PGC-1α by NAD+ dependent SIRT1 promotes mitochondrial biogenesis and ensures that the activity of PGC-1α is sensitive to both the energy and the redox balance in the cell [32]. PGC-1α co-activation of ERRα in turn promotes expression of mitochondrial SIRT3 that ensures effective scavenging of ROS at the mitochondria through activation of mitochondrial superoxide dismutase, amongst other mitochondrial sirtuin targets [43]. PARIS, a Parkin substrate, represses mitochondrial biogenesis by transcriptionally inhibiting PGC-1α expression [44].

## Mitophagy

Mitophagy is the process by which damaged or dysfunctional mitochondria are selectively engulfed by autophagosomes and delivered to lysosomes to be degraded and recycled by the cell [45]. An excess of reactive oxygen species (ROS) may function as an autophagy trigger [46] and dysfunctional mitochondria that overproduce ROS, are indeed selectively targeted for mitophagy [46].

Central to mitochondrial and cellular homeostasis, mitophagy is modulated by the PTEN-induced putative kinase 1 (PINK1)/Parkin pathway [47] which primarily targets mitochondria devoid of membrane potential (ΔΨm). PINK1 accumulate on the outer membrane of dysfunctional mitochondria and recruit the E3 ubiquitin ligase Parkin [48] [49] [50] that ubiquitinate several OMM proteins that are consequently targeted by P62/SQSTM1 [51].

P62 recognizes ubiquitinated substrates and directly interacts with autophagosome-associated LC3 to recruit autophagosomal membranes to the mitochondria [52]. Damaged mitochondria can also, independently of Parkin, increase FUNDC1 and Nix expression to recruit autophagosomes to mitochondria via direct interaction with LC3 [53] [54]. Ubiquitin ligases, like Smurf1, target depolarized mitochondria for mitophagy [55-57].

The transcription factor nuclear factor erythroid 2-related factor 2 (Nrf2) partly regulates P62 expression due to the presence of an antioxidant response element (ARE) in its promoter region [58, 59]. Electrophilic natural products such as isothiocyanate compound, sulforaphane which upregulate Nrf2 by interfering with its regulator protein, the redox sensitive ubiquitination facilitator Keap1 (Kelch-like ECH-associated protein 1) can potentially induce P62 expression [60-62]. P62-mediated mitophagy inducer (PMI) (HB229), was recently developed to upregulate P62 via stabilization of Nrf2 and promote mitophagy. This compound bypasses the upstream steps of the mitophagic cascade and acts independently of the ΔΨm collapse, and does not mediate any apparent toxic effects on mouse embryonic fibroblast (MEF) cells at the concentrations used in the assays [63]. Parkin also modulates transport of mitochondria along microtubules to a perinuclear region where autophagosomes are concentrated [64] [48]. This is likely due to Parkin-mediated turnover of Miro, a protein required to tether kinesin motor protein complexes to the OMM [65]. HDAC6, a ubiquitin-binding protein deacetylase is also recruited to mitochondria by Parkin [66] along microtubules [67, 68]. Mitophagy is crucial for cellular homeostasis and its impairment is linked to several neurodegenerative diseases [69] [70]. However, selective pharmacologic modulators of mitophagy that would facilitate dissection of the molecular steps involved in the removal of mitochondria from the network via this pathway are not presently available.

## Mitochondrial Fission

Mitochondrial fission occurs during mitochondrial biogenesis when intramitochondrial components are sorted and split into daughter mitochondria [71, 72] but also precedes the selective targeting of mitochondria for mitophagy or cellular apoptosis [73-76].

Dynamin-related protein 1 (Drp1), a member of the dynamin family of GTPases, is the major protein involved in the division of membranes through translocation from the cytosol to the outer mitochondrial membrane where constricting rings are formed [77]. While fission occurs regardless of mitochondrial membrane potential, it is upregulated following mitochondrial depolarization, oxidation or nitrosylation, and ETC inhibition which trigger posttranslational modifications, including phophorylation, S-nitrosylation, ubiquitylation, and sumoylation on Drp1[66, 78] which result in mitochondrial fragmentation [79, 80].

Drp1 lacks a pleckstrin-homology domain and requires membrane receptor proteins such as Fis1 to facilitate its association and polymerization at membranes. It can cause Bax oligomerization independent of its GTPase activity [81] while the anti-apoptotic Bcl-XL promotes mitochondrial fission in neurons through interactions with Drp1 that promote its GTPase activity [82].

In AD Aβ overproduction is associated with increased number of fragmented mitochondria, increased oxidative stress and loss of Δψ_m_ and ATP production that is associated with increased expression of Drp1 [83]. Accumulated Aβ enhances Drp1 activity in neurons by increasing Drp1 S-nitrosylation at Cys644. Likewise, AD patients are characterized by having Aβ-Drp1 mediated mitochondrial fragmentation, mtDNA mutations [84] and decrease in oxphos [85].

Mdivi-1, a small molecule noncompetitive inhibitor of Drp1 GTPase activity that attenuates Drp1 mediated mitochondrial-fission in response to pro-apoptotic stimuli [86], has been developed and may potentially have therapeutic utility. Mdivi-1 application *in vivo* has been shown to for example protect cardiomyocytes against ischemia/reperfusion injury and attenuate retinal ganglion cell death after ischemic injury [87-89]. Mdivi-1 also partially rescues the mitochondrial damage due to inactivation of PINK1 [90]. Further research targeting therapeutics aimed at preserving mitochondrial function for the treatment of disease and injury may lead to improved clinical outlook for neurodegenerative diseases such as AD.

## Mitochondrial Membrane Potential

The mitochondrial membrane potential (Δψ_m_) is created when protons are pumped from the mitochondrial matrix to the intermembrane space as electrons pass through the ETC and as a prerequisite for oxidative phosphorylation. However, the higher (more polarized) Δψ_m_, the more mtROS is generated presumably due to the slowed electron transport [91] [92] [93]. Indeed, ROS generation is decreased when Δψ_m_ is dissipated by either expressing mitochondrial uncoupling proteins (UCPs) [94] or using chemical uncouplers (Reynolds and Hastings, 1995), such as carbonyl cyanide p-(tri-fluromethoxy)phenyl-hydrazone (FCCP) [95] [96]. Small decreases in membrane potential (mild uncoupling) can reduce ROS formation by limiting the life span of reduced electron transport chain (ETC) intermediates capable of generating ROS, in addition to decreasing local oxygen tensions [97-99] [100]] without seriously compromising cellular energetics [98, 100]. However, several AD animal models, and AD patient brains show evidence of reduced ATP levels, declined complex IV activity, enhanced oxidative stress compared to controls [101] [102, 103] and decreased Δψ_m_ has been shown in AD animal models and in human cortical neurons ex vivo [102, 104, 105]. A redox-optimized ROS balance hypothesis, which states that physiological ROS signaling occurs within an optimized mitochondrial membrane potential, and oxidative stress can happen at either the extreme of high Δψ_m_ or low Δψ_m_ ([106] has been proposed to reconcile this obvious discrepancy. It is based on the fact that the redox couples involved in substrate oxidation (NADH) are closely linked to the redox couples involved in antioxidant defenses (NADPH). It is therefore vital to balance an adequate level of Δψ_m_ to maintain matrix NADPH rather than NADP^+^, which is necessary for mitochondrial antioxidant enzyme systems. This means that an increase in mitochondrial uncoupling of the ETC can increase ROS production primarily because the antioxidant system of the cell is compromised. It has now been shown that ROS can stimulate mitochondrial uncoupling [107, 108] and that the processes of uncoupling and ROS generation exist in a feedback loop [109] [108, 110].

Fatty acid (FA) cycling across the inner mitochondrial membrane is an important endogenous mild uncoupling pathway that prevents ROS release [111, 112]. In the proton-rich intermembrane space, FA anions are protonated, become uncharged and flip-flop across the inner membrane lipid bilayer. Once in the mitochondrial matrix, the proton is released and the FA anion transported back to the intermembrane space by anion carriers, which include mitochondrial uncoupling proteins [113] [114], and the adenine nucleotide translocator [112] [115, 116]. Post-ischemic tissue survival in the brain has been shown to closely correlate with uncoupling proteins expression [117].

## Mitohormesis

While ROS can generate detrimental oxidative damage, they also play a crucial role in numerous signaling and stress responses [118, 119]. Mild oxidative stress may in fact promote longevity and metabolic health through the concept of mitochondrial hormesis (mitohormesis).

Mitohormesis occurs when low levels of oxidative stress induced by either caloric restriction, exercise [120], or other stimuli trigger an adaptive response that improves overall stress resistance. This is likely via increased endogenous antioxidant defense, which eventually reduces chronic oxidative damage [121] and extends lifespan. Inhibition of glycolysis, impairment of insulin-like signaling and certain mutations in mitochondrial ETC components, are also conditions that may promote longevity via ROS-dependent mitohormesis [122]. Glucose restriction induces mitochondrial respiration and increases oxidative stress and extends *C. elegans* lifespan via the AMPK- pathway in a manner that is sensitive to the antioxidant N-acetyl cysteine, suggesting that oxidative stress is required for lifespan extension by dietary restriction [123]. Also treatment of *C. elegans* with low doses of the superoxide generating compound paraquat extend their lifespan [124]. Mild inhibition of mitochondrial respiration extends the lifespan of organisms as diverse as yeast, worms, flies and mice [125] [126] [127] presumably through ROS stimulated HIF-1 activation of gene expression that promote longevity [128]. Mild mitochondrial insults may also communicate a stress response to induce the expression of mitochondrial chaperones such as HSP-6 and HSP-60. This mitochondrial unfolded protein response (UPR^mt^) is thought to extend the life span of C. elegans by inhibiting the ETC [129, 130]. However, deletion of atfs-1, encoding for a transcription factor required for the induction of the UPR^mt^, does not avoid lifespan extension after inhibition of the ETC [129, 130], and constitutive activation of the UPR^mt^ by gain of function mutations in atfs-1 does not extend lifespan [131]. These along with complementary evidence obtained from diverse model organisms, has led to the mitohormesis model [129].

Although the evidence of mitohormesis in lifespan regulation in mammalian models is still lacking its translational implications should be considered as an ideal antioxidant therapy that prevents oxidative damage induced under pathological conditions without interfering with ROS needed for hormesis and cellular signaling.

## Caloric Restriction

Caloric restriction (CR) involves consuming 20-40% lower calories than normal has been suggested as a promising intervention to increase both median and maximal lifespan in humans (Peterson et al, 2012). It can prevent or delay several diseases including cancer, cardiovascular diseases, neurodegenerative disorders, diabetes and autoimmune diseases [132] and has been reported to protect against age-related mitochondrial dysfunction [133] and reduce mtDNA damage [134]. In animal models of neurodegenerative diseases it promotes neurogenesis and enhances synaptic plasticity [135], improves cognitive capability, anti-inflammatory mechanisms, reduce neural oxidative stress, induce various stress and neurotrophic/neuroprotective factors and prevents Aβ neuropathology in AD transgenic models [136]. At the cellular level, CR alters Δψ_m_ and respiratory activity, which results in lower ROS generation and oxidative damage. CR also increases mitochondrial biogenesis and bioenergetic efficiency through Akt, which directly phosphorylates and activates endothelial nitric oxide synthase (eNOS) leading to nitric oxide (NO) production [137, 138] [139] [140, 141] [142]. NO activates a NO/cGMP-dependent signaling pathway that induces PGC-1α, increasing mitochondrial biogenesis [143, 144]. This increase in mitochondrial biogenesis elicits the beneficial effects of CR [138, 139, 142, 143]. Mice on 3 months CR have higher levels of mitochondrial DNA, PGC-1α, NRF-1, Tfam, expression of cytochrome c oxidase, and cytochrome c when compared with mice fed ad libitum, indicating increased mitochondrial biogenesis [139]. 2,4-dinitrophenol, a CR mimetic induces similar results [142]. Long term exposure to elevated ROS levels impairs eNOS activity [145, 146]. As a result eNOS functions in a negative feedback loop preventing the generation of excessive ROS. In a CR trial CALERIE based on 25% CR, CR patients were shown to have less mtDNA damage, more mtDNA content, and increased expression of some antioxidant enzymes, suggesting that CR improves mitochondrial function and delays mitochondrial aging through reducing oxidative stress. The increase in expression of several proteins involved in mitochondrial biogenesis such as PGC-1*α*, Tfam, and SIRT1 was reported in CR patients compared to controls [147]. CR also attenuates the age-related decline of autophagy by activating Sirt1 which deacetylates autophagy proteins [148-150] and this is associated with increased longevity as inefficient mitochondria are replaced with new functional mitochondria [151, 152]. The net result is that CR reduces oxidative stress and enhances mitochondrial biogenesis in order to produce mitochondria that are more efficient in ATP production, have optimal oxidative capacity, and generate less ROS.

## Exercise

Exercise training alone or in combination with CR may also represent an efficient strategy to delay mitochondrial aging and age-related dysfunction in humans through mechanisms stimulating mitochondrial biogenesis and oxidative capacity and improving protein quality control [153]. Skeletal muscle biopsies of humans performing high- intensity interval training showed an increase in Sirt1, nuclear PGC-1α and Tfam, which lead to an increase in skeletal muscle mitochondria and improved exercise performance [154-156]. Biopsies performed in older men showed that even with aging, exercise increases mitochondrial DNA and mitochondrial respiratory chain activity which is likely related to increases in mitochondria biogenesis [157, 158]. While exercise training optimized mitochondrial function in elderly individuals [159] [160] when combined with a low carbohydrate (glycogen) diet increases the expression of PGC-1*α* to optimize the oxidative capacity of human skeletal muscle [161]. In the CALERIE trial, CR with exercise training resulted in a 38% reduction in the estimated risk of cardiovascular disease, an important age-associated pathology, compared to controls [162]. Indeed, increased physical activity or even simply adopting active style habits may clearly reduce the rate of mitochondrial decline and attenuate the age-related phenotype. This exercise-induced increase in mitochondrial biogenesis is mediated through ROS as demonstrated by oral administration of antioxidants to rats impairs the exercise-induced increase in mRNA and protein levels of PGC-1α, NRF-1 and Tfam and cytochrome c [163]. Similar observations are made in humans. The exercise induced increase in PGC-1α and PGC-1β ameliorate insulin resistance and initiate an adaptive response promoting endogenous antioxidant defense capacity. However, when the subjects diet was supplemented with antioxidants these effects were not observed [120]. Importantly, it was found that exercise training also increases brain mitochondrial biogenesis (mtDNA, and PGC-1α, SIRT1, and citrate synthase) and this may have important implications, not only with regard to fatigue, but also with respect to various central nervous system diseases and age-related dementia that are often characterized by mitochondrial dysfunction [153]. Therefore, exercise could be considered as a therapeutic option to reduce the negative effects of aging and decrease the risk of AD.

## Mitochondrial Stress Response Signaling

Mitochondria are the major source of cellular ROS and hence stress signaling that induces cellular senescence and apoptosis [118] [164] [165, 166] [167]. One of the major consequences of increased ROS and altered cellular redox state is the oxidation of thiol groups in cysteine residues in relevant proteins [118]. FoxO transcription factors are activated in response to elevated ROS levels to induce anti-oxidant responses (increased expression of catalase and SOD2), cell cycle arrest and/or cell death [168, 169]. Kinases that modulate cellular stress responses include mitochondrial Akt, GSK-3β, PKA, Abl, PKC, Src and Atm [170] [171-177]. Akt phosphorylates and inactivates GSK-3β, which can localize to the mitochondria. Mitochondrial GSK-3β phosphorylates MCL-1 and VDAC [174, 178] Sheldon et al., 2011) leading to MCL-1 degradation and inducing apoptosis [178]; while phosphorylation of VDAC by GSK-3β results in increased mitochondrial membrane permeability, again predisposing to apoptosis [174, 179]. GSK-3β is also known to phosphorylate and promote the proteasomal degradation of c-Myc, cyclin D1, and β-catenin [180, 181] [182]. PKA can be translocated to mitochondria by hypoxia and other physiological stresses [183, 184] where it associates with the mitochondria through Rab32 and other A-kinase AKAPs [171] and phosphorylates VDAC [172], Drp1 [183], and other mitochondrial proteins. For example, hypoxia destabilizes AKAP121 through induction of SIAH2, a mitochondrial ubiquitin ligase, thereby limiting oxidative capacity under conditions of low oxygen. Interestingly, AKAP121 also appears to promote mitochondrial localization of Src-tyrosine kinase [185] where Src appears to regulate CO activity and respiratory activity [185] [186], and other mitochondrial substrates for Src family kinases are likely [187]. Increased ROS induces protein kinase C-delta (PKCδ) association with the mitochondria and this in turn recruits other signaling molecules, including the Abl tyrosine kinase that is associated with loss of membrane potential and non-apoptotic cell death [175]. Impaired oxidative metabolism and decreased ATP levels in neurons activate AMPK [188]. AMPK can also be activated by drugs such as metformin that inhibits complex I or resveratrol that inhibits the F0F1 ATPase [170]. AMPK modulates mitochondrial metabolism and targets Acetyl CoA carboxylase-2 (ACC2) to the OMM where it regulates lipid metabolism by controlling production of malonyl CoA [170]. AMPK therefore plays a key role in mitochondrial homeostasis by ensuring that only functionally viable mitochondria are retained. Upon its activation it induces not only mitochondrial biogenesis through activation of PGC-1α [189, 190] but also initiates mitophagy through ULK1 activation and mTOR inhibition [183, 191]. ATM kinase inhibition has been shown to cause CNS neurodegeneration in animal models [192]. ATM kinase, which is partly located at the mitochondria, is activated upon mitochondrial uncoupling [193] and while its mitochondrial substrates are not known, loss of ATM in genetically engineered mouse models leads to mitochondrial dysfunction.

**Figure 1.**
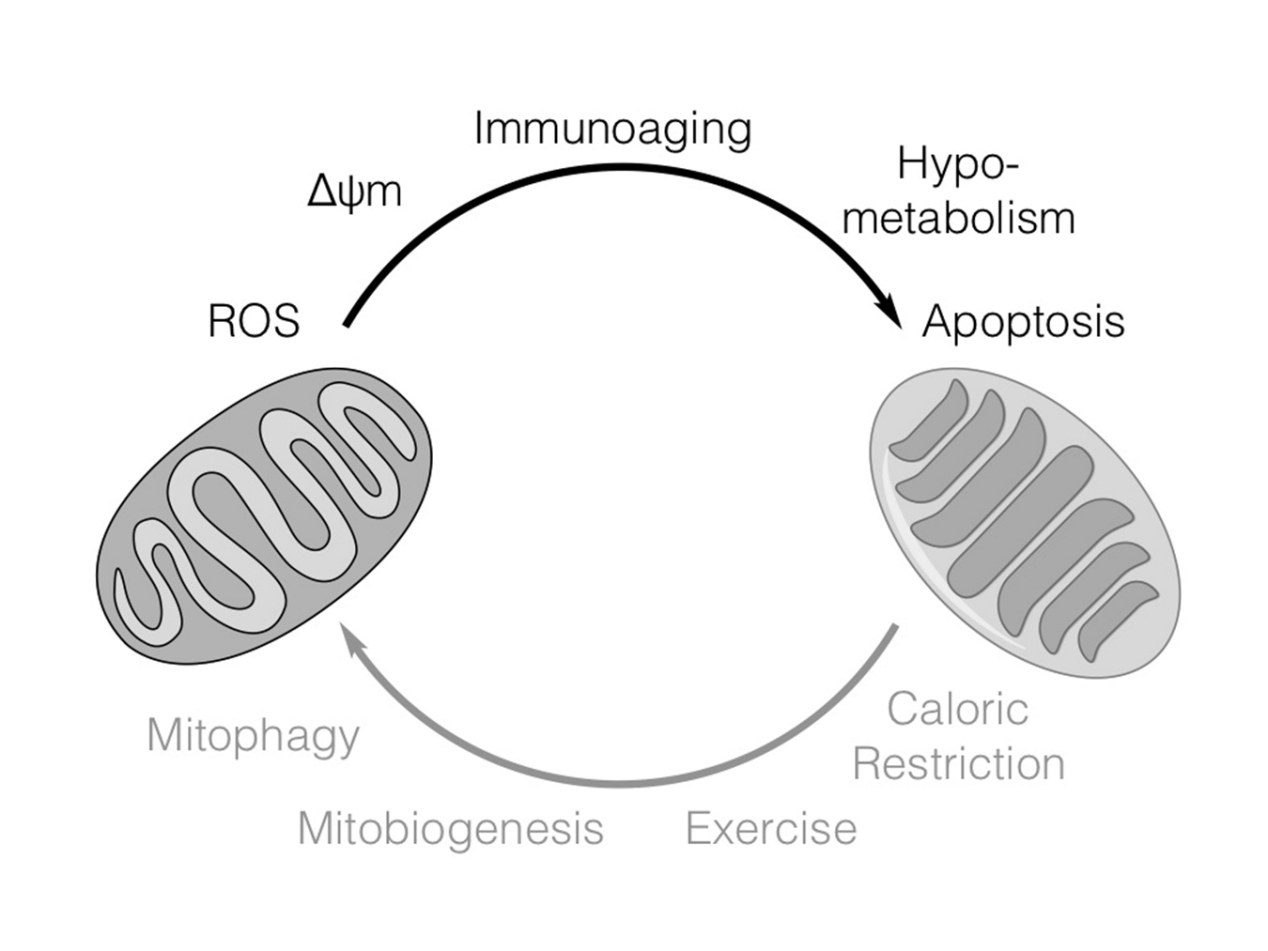
**Factors regulating mitochondrial function in AD**. In AD, neuronal injury, inflammation and aging may impair mitochondrial function by inducing fission, increasing ∆ψm and ROS production leading to decreased ATP production. Mitochondrial function may be improved by enhancing mitochondrial biogenesis through caloric restriction and exercise. Damaged and dysfunctional mitochondria can be selectively eliminated by mitophagy.

## Immunoaging and Mitochondrial Function

Immune function is compromised during the course of aging as well as in AD. Investigations of changes in adaptive immune function associated with aging indicate disturbances of T- and B-cell homeostasis and activation as well as that of marcophages [194, 195]. Large scale (BLSA and MESA) transcriptomic studies of CD4+ T-cells and CD14+ monocytes from aged individuals show mitochondrial pathways, particularly OxPhos, as the most down-regulated (FDR <0.001) [196, 197]. The loss in OxPhos expression is significant as mitochondrial oxidative metabolism plays a critical regulatory role in immune function. Mitochondria serve as the scaffold for NLRP3 inflammasome formation, where mitochondrial ROS and oxidative metabolism regulate caspase-1 activation, the critical step in maturation of Il-1beta and Il-18. Mitochondrial oxidative metabolism regulates macrophage polarization, T-cell activation, differentiation and memory cell formation (for review see Weinberg et al., 2015 [198]). Thus, mitochondria not only sustain immune cell phenotypes but also are necessary for establishing immune cell phenotype and function. In a pro-inflammatory state this is accomplished by mitochondria shifting from producing ATP via oxidative metabolism to producing building blocks for macromolecule synthesis via anapleurosis and glutaminolysis. The shift from catabolism to anabolism is critical to affect cell expansion, production of inflammatory mediators and immune cell fate commitments. This may explain why the increase in serum pro-inflammatory cytokines occurs with age, giving rise to a chronic state of inflammation, termed inflammaging [199-201].

In AD, immune dysfunction has been identified in T- and B-cells, macrophages and microglia [202]. AD is associated with increased T cell infiltration, changes in immune populations associated with disease progression, reduction in T- and B-cell numbers and reductions in CD4+CD25+ Tregs [203]. CD8+CD28- suppressor cells are also decreased in PBMCs from AD patients. These data suggest that the immunosuppressive capabilities in AD patients are diminished and could represent a deficit in the ability to control Teff responses. As such, increased activities of Th17, levels of IL-21, IL-6, and IL-23, and the Th17-associated transcription factor RORγ, were increased among lymphocytes in AD patients [204]. This suggests AD specific overactivity of Th17 T-cell function and underactivity of Teff function. Given that Th17 T-cells primarily mobilize glycolysis and suppress OxPhos whereas Tregs and memory T cells oxidize fatty acids via mitochondrial oxidation, supports the concept that mitochondrial dysfunction fuels AD immune dysfunction [205].

## Conclusion

Mitochondrial function is deregulated in AD and there is growing interest in understanding how altered mitochondrial function may be targeted to inhibit neurodegeneration. Proper modulation of mitochondrial turnover overall to eliminate dysfunctional mitochondria while maintaining efficient functional mitochondrial mass in response to stresses, including hypoxia and nutrient starvation may be relevant in delaying or managing the degenerative process in aging and AD. By preventing the generation of excessive ROS and conserving valuable nutrients neuronal survival may be promoted under conditions of energetic stress (Figure 1).
